# Spatial patterns of new leprosy cases in a northeastern state of Brazil, 2011–2021

**DOI:** 10.1590/1980-549720230014

**Published:** 2023-02-20

**Authors:** Maria Luiza Ferreira Imburana da Silva, Shirlley Jackllanny Martins de Farias, Amanda Priscila de Santana Cabral Silva, Maria Olívia Soares Rodrigues, Emília Carolle Azevedo de Oliveira

**Affiliations:** I Secretaria de Saúde do Recife, Programa de Residência Multiprofissional em Vigilância em Saúde – Recife (PE), Brazil.; II Universidade Federal de Pernambuco – Vitoria de Santo Antão (PE), Brazil.; III Fundação Oswaldo Cruz, Instituto Aggeu Magalhães – Recife (PE), Brazil.; IV Agência Pernambucana de Vigilância Sanitária – Petrolina (PE), Brazil.

**Keywords:** Leprosy, Spatial analysis, Neglected diseases, Epidemiology, Hanseníase, Análise espacial, Doenças negligenciadas, Epidemiologia

## Abstract

**Objective:**

To analyze the spatial patterns of leprosy in Pernambuco from 2011 to 2021.

**Methods:**

This is an ecological epidemiological study, carried out with data from the Notifiable Diseases Information System, based on new cases of leprosy among inhabitants of Pernambuco, between 2011–2021. An empirical Bayesian analysis of local and spatial dependence was performed with the global and local Moran indices.

**Results:**

25,008 new cases of leprosy were registered with an annual case detection rate in the general population of 16.51 cases/100,000 inhabitants — which is considered high. Among those younger than 15 years of age, there were 5.16 cases/100,000 inhabitants (high) and 0.89/100,000 inhabitants with degree II of physical disability (low); there were also many high-risk cases with an overall Moran index of 0.33 (p<0.001), active transmission (0.26; p<0.001), and subsequent diagnosis of the disease (0.12; p<0.006), as well as distribution in macro-region 1 and macro-region 4.

**Conclusion:**

There was a heterogeneous spatial distribution in the state, showing two overviews, the first being the presence of municipalities with high risk of disease transmission and the second with clusters of silent municipalities, reinforcing the character of leprosy neglect as a major public health problem. This study brings reflections for leprosy control actions, due to the identification of priority areas to combat this disease in Pernambuco.

## RESUMO

**Objetivo:**

Analisar os padrões espaciais da hanseníase em Pernambuco no período de 2011 a 2021.

**Métodos:**

Trata-se de um estudo epidemiológico ecológico, realizado com dados do Sistema de Informações de Agravos de Notificação com base nos casos novos de hanseníase em residentes de Pernambuco, entre 2011–2021. Foi realizada uma análise bayesiana empírica local e de dependência espacial com os índices de Moran global e local.

**Resultados:**

Foram registrados 25.008 casos novos de hanseníase com coeficiente médio anual de detecção de casos novos na população geral de 16,51 casos/100 mil habitantes — classificado como alto. Na população menor de 15 anos, ocorreu 5,16 casos/100 mil habitantes (muito alto) e 0,89/100 mil habitantes com grau II de incapacidade física (baixo) com aglomerados de alto risco para ocorrência com índice global de Moran de 0,33 (p<0,001), transmissão ativa (0,26; p<0,001) e diagnóstico tardio da doença (0,12; p<0,006), localizados na macrorregião 1 e na macrorregião 4.

**Conclusão:**

Houve uma distribuição espacial heterogênea no estado, evidenciando dois panoramas, o primeiro, a presença de municípios com alto risco de transmissão da doença e, o segundo, aglomerados de municípios silenciosos reforçando o caráter de negligência da hanseníase como grande problema de saúde pública. Este estudo traz reflexões para ações de controle da hanseníase, dada a identificação de áreas prioritárias para o enfrentamento dess\a doença em Pernambuco.

## INTRODUCTION

Leprosy is an infectious and contagious disease, caused by the bacillus *Mycobacterium leprae*, which manifests itself mainly in the skin and peripheral nerves and can cause physical disabilities and deformities^1^. Despite efforts to eliminate the disease, it continues to occupy second place in the world ranking of morbidity, affecting mainly developing countries^2,3^.

In 2017, Brazil registered 26,875 cases, second only to India, with 126,164 cases^4^, and had an overall detection coefficient of 12.94 cases/100,000 inhabitants, considered highly endemic^5^. The presence of clusters of new cases of leprosy in different Brazilian states indicates the maintenance of the high burden of the disease in delimited areas^6^. According to the World Health Organization (WHO), in the last ten years, around 30 thousand new cases were registered per year in Brazil.

In the Brazilian ranking, Pernambuco occupies the 7^th^ place in the detection coefficient in children under 15 years of age and the 8^th^ place in the general detection coefficient. In the Northeast Region, the state occupies the 3^rd^ place in both coefficients — general detection and in children under 15 years of age. In this context of active transmission and high detection rates, actions to control the disease are a priority in Pernambuco^7,8^.

Spatial analysis is a tool used to identify the distribution of leprosy in the territory, in order to strengthen control strategies in certain geographic areas, being considered as a guide for intervention in high-risk areas^9^.

Thus, knowing the spatial patterns of a disease in a given region is essential to plan surveillance and control actions. In view of the above, the objective of this study is to analyze the spatial patterns of leprosy in Pernambuco from 2011 to 2021.

## METHODS

This is an ecological study with spatial analysis of a quantitative nature, consisted of new cases of leprosy among residents of Pernambuco, notified in the period from 2011 to 2021. The databases used were: Information System for Notifiable Diseases (*Sistema de Informação de Agravos de Notificação* – SINAN), Department of Informatics of the Unified Health System of Brazil (*Departamento de Informática do Sistema Único de Saúde do Brasil* – DataSUS), and the Brazilian Institute of Geography and Statistics (*Instituto Brasileiro de Geografia e Estatística* – IBGE).

**Figure 1:**
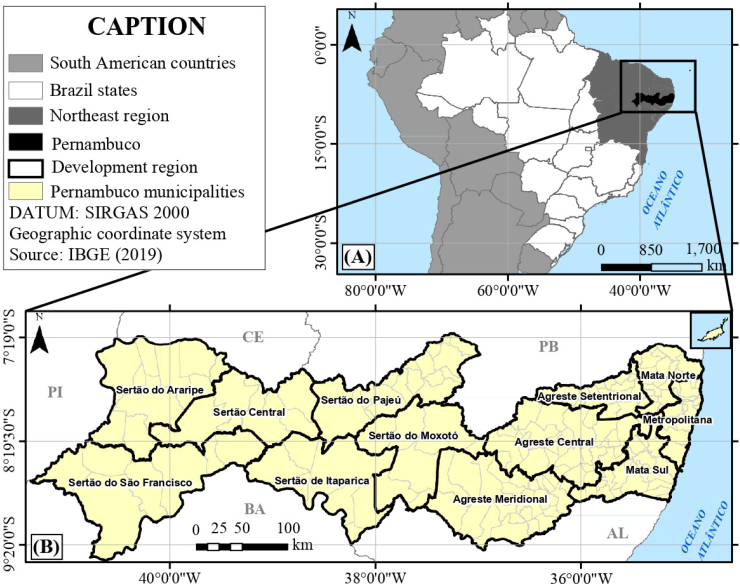
Location of the study area: (A) map of Brazil highlighting Pernambuco; (B) state of Pernambuco.

Source: Own elaboration based on data from the Brazilian Institute of Geography and Statistics (*Instituto Brasileiro de Geografia e Estatística* – IBGE).

The state of Pernambuco is located in the Northeast of Brazil and is bordered to the north by Paraíba and Ceará, to the east by the Atlantic Ocean, to the west and south by Piauí and Bahia and to the south by Alagoas. It is divided into 184 municipalities, in addition to the district of Fernando de Noronha, and has a territorial extension of 98,146.315 km^2^. As a regionalization strategy, the state is divided into 12 development regions (Figure 1) and 12 health regions. It is the seventh most populous state in the country, with an estimated population of 9,376,936 in 2016 and a population density of ~95 inhab/km^2^.

For the spatial analysis, three indicators stipulated by the WHO were selected:

1. The coefficient of detection of new cases in the general population (per 100 thousand inhabitants), which reveals the magnitude of the disease;
2. The detection coefficient in children under 15 years of age (per 100,000 inhabitants), which demonstrates the active transmission of leprosy; and
3. The number of new cases with grade II disability detected in a population (per 100,000 inhabitants), which reflects late diagnosis and underdetection of the disease.

The descriptive analysis comprises the mean, minimum, maximum, and standard deviation statistics. Subsequently, the coefficients were classified as recommended by the WHO (Chart 1).

**Chart 1:** Description of leprosy detection coefficients in the general population, in children under 15 years of age and with grade II leprosy deformity.

Coefficient		Value
Detection coefficient of the general population (thousand inhabitants)		
	Low	<2.00 per 100
	Medium	2.00 to 9.99 per 100
	High	10.00 to 19.99 per 100
	Very high	20.00 to 39.99 per 100
	Hyperendemic	≥40.00 per 100
Detection coefficient <15 years (thousand inhabitants)		
	Low	<0.50 per 100
	Medium	0.50 to 2.49 per 100
	High	2.50 to 4.99 per 100
	Very high	2.50 to 4.99 per 100
	Hyperendemic	≥10.00 per 100
	Very hyperendemic*	>20.00 per 100
Grade II detection coefficient (thousand inhabitants)		
	Low	<2.00 per 100
	Medium	2.00 to 4.99 per 100
	High	5.00 to 9.99 per 100
	Very high	>10.00 per 100

* Classification by Monteiro et al.^2^

Source: Own elaboration.

The percentages were calculated taking into account all municipalities in Pernambuco, and not just those that had new cases during the period studied.

The coefficients were calculated by the median of the population, which is equivalent to the estimate for the year 2016 to correct randomness and obtain greater stability in municipalities with small populations (less than 20,000 inhabitants). The indicators were smoothed by the local empirical Bayesian method, which uses information from neighboring areas when estimating the risk of the area^10^.

In order to verify the global spatial autocorrelation, after the descriptive analysis, the global Moran test was applied to obtain the coefficient that measures the spatial autocorrelation of a variable in areas, ranging from -1 to +1. The global Moran coefficient with values closer to zero indicate the absence of spatial autocorrelation; positive values, in turn, indicate the existence of positive autocorrelation; and when the index shows negative values, it means that there is a negative (inverse) autocorrelation.

In general, the Moran index can be subjected to a test whose null hypothesis demonstrates spatial independence [I=0]. So, the null hypothesis can only be rejected if [I] results statistically different from zero and reaches a pre-established significance level^11^. In this study, a confidence margin greater than 95% was used [p-value<0.05].

To identify significant spatial groupings or clusters, the Local Indicator of Spatial Association (LISA) was calculated and the test was performed considering 10% of significance and 99 permutations^12^.

Based on LISA values and deviations, municipalities were classified into four quadrants:

Q1, high/high (above-average municipality with above-average neighbors);

Q2, low/low (below-average municipality with below-average neighbors);

Q3, high/low (above-average municipality with below-average neighbors); and

Q4, low/high (below-average municipalities with above-average neighbors).

Therefore, thematic maps were generated indicating which quadrant each municipality belongs to, considering the municipalities with statistically significant differences (p<0.1), called in the literature by Moran Map or cluster map.

Spatial analysis was carried out by preparing distribution maps of epidemiological indicators, using the software QGIS, Version 3.26.1, and TerraView Version 4.2.2, using the mesh of municipalities on the IBGE website.

This study used secondary data from the public domain. In this sense, by item III of Resolution 510/2016, there was no need for the project to be assessed by the research ethics committee^13^.

## RESULTS

In the period from 2011 to 2021, 25,008 new cases of leprosy were registered in Pernambuco. The mean annual coefficient of detection of new cases in the general population was 16.51 cases/100,000 inhabitants, being classified as high. In the population under 15 years of age, 5.16 cases/100,000 inhabitants were diagnosed, which is very high, and 0.89 cases/100,000 inhabitants with grade II of physical disability, classified as low (Chart 2).

**Chart 2:** Descriptive analysis of leprosy detection coefficients in the general population, in children under 15 years old and with grade II leprosy deformity in Pernambuco, 2011 to 2021*.

Coefficient	Mean	Minimum	Municipality (minimum)	Maximum	Municipality (maximum)	Standard deviation
Detection coefficient of the general population	16.51	1.44	Carnaubeira da Penha	99.71	Trindade	±15.02
Detection coefficient <15 years	5.16	0.00	51 municipalities (27.57%)	40.25	Itapissuma	±6.82
Grade II detection coefficient	0.89	0.00	47 municipalities (25.41%)	5.22	Trindade	±0.95

* Data subject to change.

Source: Own elaboration based on data from the Notifiable Diseases Information System (*Sistema de Informação de Agravos de Notificação* – SINAN).

It was observed that new cases of leprosy occurred in 100% of the municipalities, 72.43% of these had at least one case of the disease in children under 15 years of age, and 74.59% had at least one case with grade II physical disability.

Still, as shown in Chart 2, the municipality of Carnaubeira da Penha obtained the lowest mean detection coefficient of the general population (1.44/100,000 inhabitants), being classified as low. On the other hand, the municipality of Trindade had the highest detection coefficient, with a mean of 99.71/100,000 inhabitants, classified as hyperendemic and causing a wide range of data.

The detection coefficient for <15 years reached a minimum of zero (no cases registered) for 51 municipalities and a maximum for the municipality of Itapissuma, located in the metropolitan region, with the classification “very hyperendemic”. The degree II detection coefficient obtained a minimum of zero (no cases registered) for 47 municipalities and a maximum for the municipality of Trindade, with a high classification.

**Figure 2:**
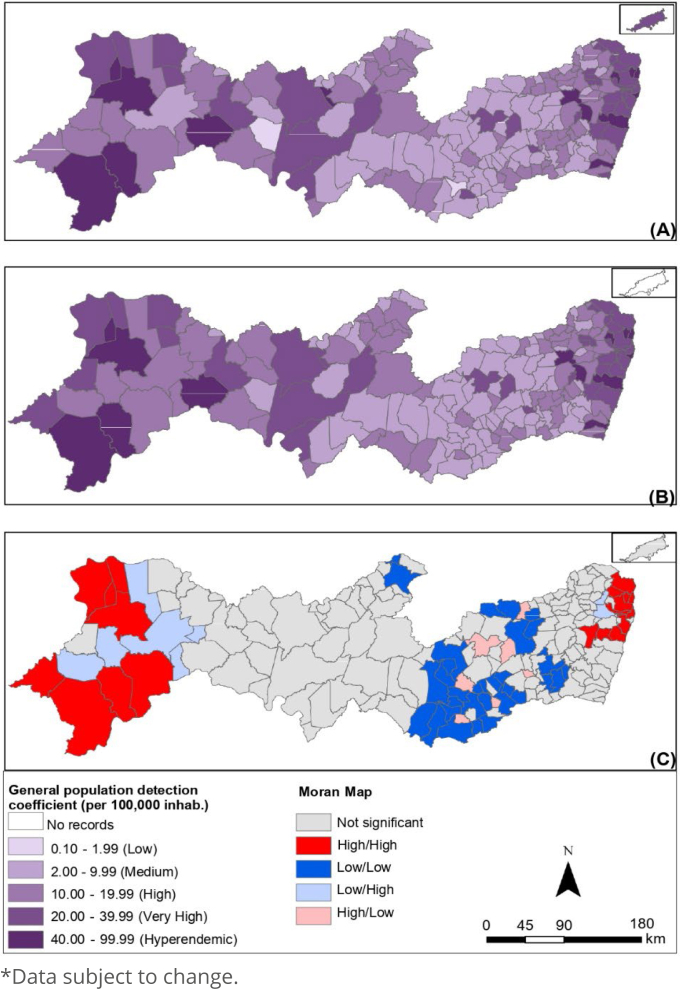
Spatial analysis of the detection coefficient of new cases of leprosy in the general population (per 100,000 inhabitants): gross coefficient (A), coefficient smoothed by the local empirical Bayesian method (B), and Moran Map (C). Pernambuco, 2011-2021*.

Source: Own elaboration based on data from the Notifiable Disease Information System (*Sistema de Informação de Agravos de Notificação* – SINAN) and the Brazilian Institute of Geography and Statistics (*Instituto Brasileiro de Geografia e Estatística* – IBGE).

**Figure 3:**
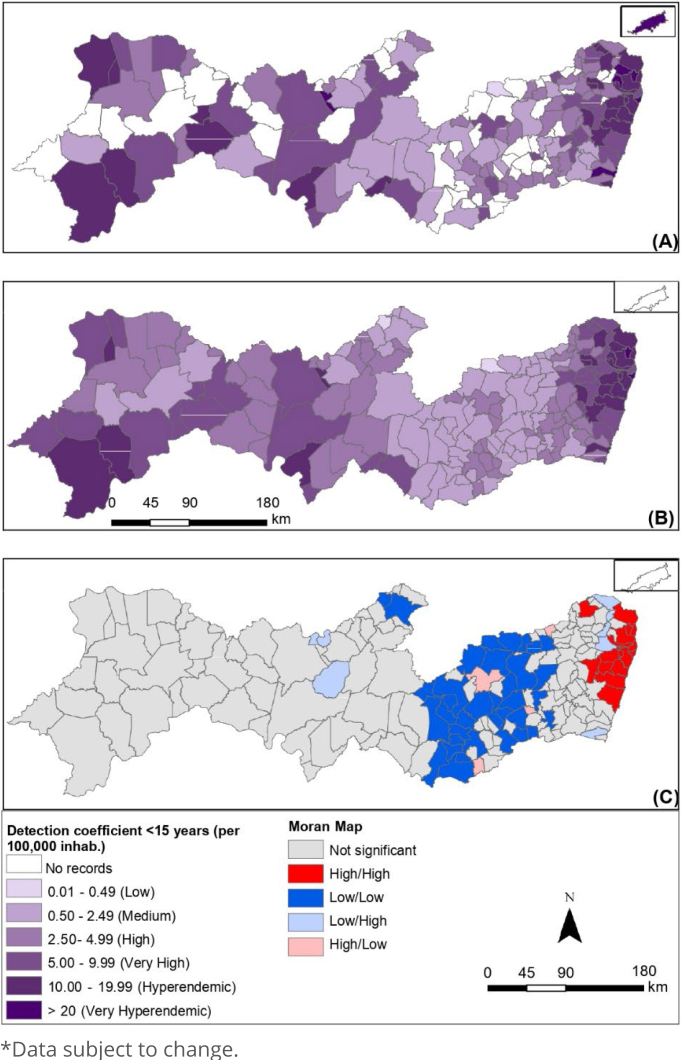
Spatial analysis of the detection coefficient of new cases of leprosy in children under 15 years of age (per 100,000 inhabitants): gross coefficient (A), coefficient smoothed by the local empirical Bayesian method (B) and Moran Maps (C). Pernambuco, 2011-2021*.

Source: Own elaboration based on data from the Notifiable Disease Information System (*Sistema de Informação de Agravos de Notificação* – SINAN) and the Brazilian Institute of Geography and Statistics (*Instituto Brasileiro de Geografia e Estatística* – IBGE).

**Figure 4:**
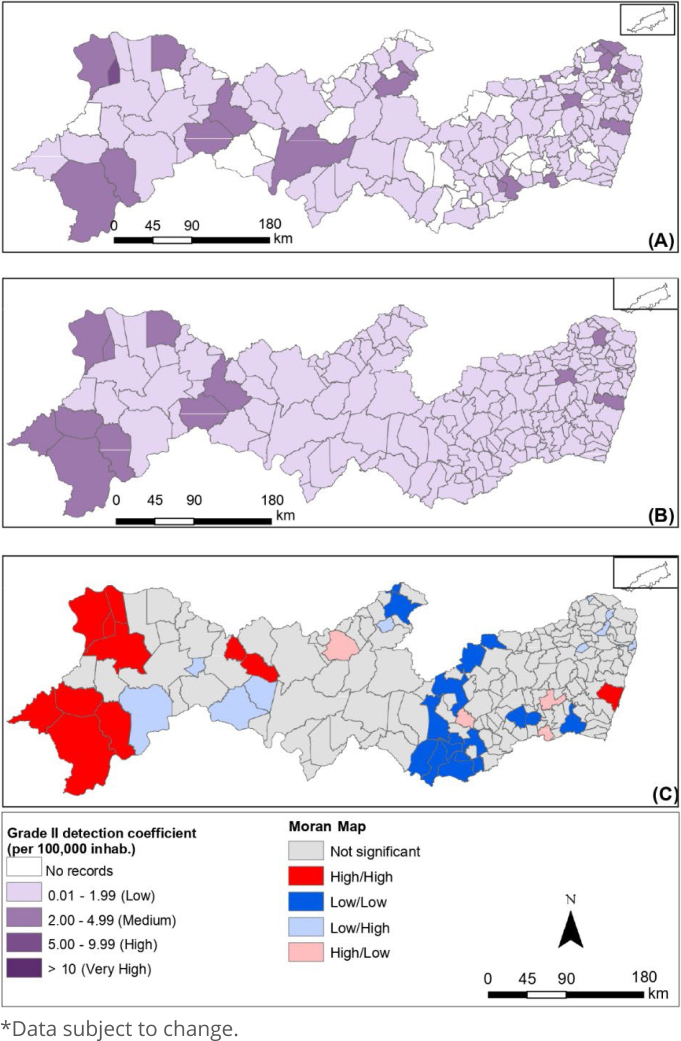
Spatial analysis of the detection coefficient of new cases of leprosy with degree II of physical disability (per 100,000 inhabitants): gross coefficient (A), coefficient smoothed by the local empirical Bayesian method (B) and Moran Map (C). Pernambuco, 2011-2021*.

Source: Own elaboration based on data from the Notifiable Disease Information System (*Sistema de Informação de Agravos de Notificação* – SINAN) and the Brazilian Institute of Geography and Statistics (*Instituto Brasileiro de Geografia e Estatística* – IBGE).

Comparing the standard deviation of the three coefficients, the detection coefficient for the general population showed greater variability between the data, followed by the detection coefficient <15 years and the degree II detection coefficient, which showed less variability, as shown in Figures 2A, 3A and 4A, respectively, in the state of Pernambuco.

For the detection coefficient of the general population (Figure 2A), 52.43% (n=97) of the municipalities have high and very high coefficients, 7.57% (n=14) are in a hyperendemic situation. Only 1.08% (n=2) have a low coefficient and 38.92% (n=72) have a medium coefficient.

It is also possible to identify a concentration of the highest coefficients in the metropolitan regions (37.26/100 thousand inhab.), Sertão do São Francisco (37.02/100 thousand inhab.), and Sertão do Araripe (32.94/100 thousand inhab.), all considered very high rates. On the other hand, the lowest rates are concentrated in the middle of the state, specifically in the development regions of Agreste Meridional (7.83/100,000 inhab.), Agreste Central (10.86/100,000 inhab.), and Sertão do Moxotó (11.61/100 thousand inhab.).

The leprosy detection coefficient in children under 15 years of age (Figure 3A) shows 3.78% (n=7) of the municipalities with a coefficient considered very hyperendemic, 14.05% (n=26) hyperendemic, and almost 40% (n =72) among municipalities considered to have high to very high rates. This coefficient has the worst scenario in relation to the WHO classification, being hyperendemic in the metropolitan region, with a mean coefficient of 18.56/100,000 inhab., where 71.43% of all hyperendemic municipalities are concentrated.

Only 15.68% (n=29) of the municipalities have a detection coefficient for leprosy in children under 15 years of age with medium and low parameters, and 27.57% (n=51) of the municipalities did not register new cases during the study period.

For the degree II detection coefficient (Figure 4A), 11.89% of the municipalities have medium coefficients, with a gross coefficient of values between 0.01-1.99 (low) in most of the state, corresponding to 62.70% coverage of the study area. Of the municipalities studied, 25.41% had no case records, a value that reflects the lack of new cases for the degree II detection coefficient, as well as the small quantity compared to the exposed population.

Figures 2B–C, 3B–C and 4B–C correspond to the raw coefficients smoothed by the Moran spatial statistics and the Moran Maps generated based on the smoothed rate, respectively.

These results were only possible as the global Moran index was statistically significant (p-value<0.05) for the three coefficients. The Moran Global Index confirmed the existence of spatial dependence between municipalities, the general population detection coefficient (0.33 I-Global; p=0.001), the detection coefficient <15 years (0.26 I-Global; p=0.001), and the degree II detection coefficient (0.12 I-Global; p=0.006).

The Sertão do São Francisco, Sertão do Araripe, and metropolitan regions presented municipalities with a high/high standard for the detection coefficient of the general population; municipalities in the metropolitan region had a high/high pattern in the detection coefficient <15 years; and the regions of Sertão do São Francisco and Sertão do Araripe with municipalities with a high/high pattern in the degree II detection coefficient. The other regions of the state had municipalities with a low/low pattern for the three epidemiological indicators.

## DISCUSSION

In this research, it was observed that the patterns of leprosy in the period studied showed significant spatial heterogeneity, with high endemicity in Pernambuco and variations in the detection coefficient between municipalities, with some being hyperendemic^14^. It is also noted the proximity of municipalities with high leprosy detection rates to those with low rates.

Marquetti et al.^15^ claim that there is a relationship between high rates and low levels of socioeconomic development, high social and spatial vulnerability of people affected by leprosy. Thus, the high rates found in the present study may be related to the context of vulnerability in which the regions are immersed.

Considering that Pernambuco presents several conditions of social inequalities, with more than 40% of the population below the poverty line, approximately 30% of individuals aged 14 years old or older had informal work, in addition to occupying the 17^th^ place among Brazilian states in the ranking of Municipal Human Development Index (HDI-M), with a value of 0.727^15–18^.

Another probable explanation for the high leprosy rates is the actions developed by the state control programs and the expansion of the primary health care network in Brazil in recent years, whose work increased the detection of new cases. However, its expansion does not express more access to services, as care conditions are still unsatisfactory for early diagnosis and treatment of the disease^15,17^. It is necessary to consider the performance of the units, the flows and work processes, the infrastructure, and the availability of trained professionals for this service^19^.

In addition to the unsatisfactory conditions of health services that increase programmatic vulnerability, other social determinants contribute to the maintenance of leprosy, such as income, housing, and education^20^. Some studies^20–22^ have pointed to a positive association between leprosy and low educational level, low levels of income and schooling, with the majority of the population considered poor or indigent and not users of assistance benefits, reaffirming the contexts of vulnerability of leprosy as a serious public health matter^20^.

Therefore, aspects related to social vulnerability need to be better understood in the persistence of leprosy, to help plan actions to combat the disease, promoting the reduction of social and health inequalities, in addition to research on new indicators associated with surveillance of contacts^22^. In addition, it is important to emphasize the importance of socio-educational activities to increase individuals’ knowledge about the infectiousness of the disease, its transmission, and treatment^20^.

When analyzing the statistically significant areas with the Moran Map, it was observed that the clusters of the high-high type in relation to the detection rate of the general leprosy population were concentrated in three regions: metropolitan, Sertão do São Francisco, and Sertão do Araripe. This epidemiological indicator reflects the burden of morbidity and the magnitude of the disease, estimating the risk of occurrence of new cases in any clinical form, thus indicating exposure to the bacillus Mycobacterium leprae *Mycobacterium leprae* ^17^.

The case detection rate in children under 15 years of age identified clusters of the high-high type with the Moran Map in the metropolitan region, indicating the presence of leprosy and its recent transmission force with active transmission foci in the family or among contacts^23^. The identification of the main highly endemic clusters in the metropolitan region corroborates the findings of another study^24^, thus confirming the urban nature of the endemic disease^14^, considering that the process of urbanization and geographic expansion can help in the circulation and maintenance of leprosy in the region^25^.

The degree II disability detection coefficient prevails with low and medium rates, with significant clusters on the Moran Map in the Sertão do São Francisco and Sertão do Araripe regions, data similar to that identified in the detection rate of the general population, thus corroborating another survey^14^.

Still, this aforementioned coefficient indicates late diagnosis with complications, hidden prevalence and possible problems in the diagnosis that may have obstacles given the similarity of symptoms with skin diseases and neuropathic problems, as well as signals that there are problems in the early treatment of leprosy^2^.

The metropolitan regions, Sertão do São Francisco, and Araripe are located at the two extremes of the state, therefore, they have different socioeconomic characteristics. The first is considered the most developed, made up of 16 municipalities, where most of the population is concentrated. In 2021, it had 4,050,233 inhabitants and, in 2019, it had a demographic density of 1,461.94 inhabitants/km^2 26^. This region, which has the highest gross domestic product *per capita* (R$ 38,455.45), it is also the region with the greatest social inequality, in which 42.28% of its population has an income of less than half a minimum wage^26,27^. This situation reinforces the hypothesis that leprosy is a condition that more often affects people with social disadvantages.

In addition, the metropolitan region is a reference in health services in the state, as it has the highest density of highly complex services and health surveillance structure in Pernambuco. Furthermore, due to its history of higher detection rates for new cases of leprosy, it has the highest number of priority municipalities for the Sanar program, which includes leprosy in the group of priority diseases for control in the state^28^.

Unlike the metropolitan region, the regions of Sertão do São Francisco and Araripe make up the same macro-region of health and are formed by municipalities that are farther from the capital and with a lower rate of urbanization^29^. The Sertão do São Francisco region has 517,029 inhabitants, while the Araripe region has 337,916 inhabitants.

Like the first region, the others also present a scenario of socioeconomic vulnerability, which reaffirms the relationship between low socioeconomic conditions and high detection rates of leprosy, since, in the regions of São Francisco and Araripe, the percentage of the population that survives on an income of less than half the minimum wage is 54.57 and 70.22%, respectively^26,27^. The municipality with the highest detection rate in the state was Trindade, which is located in the Araripe region and has a population of 26,116 inhabitants (2021 data) and an HDI of 0.595, below the state rate^30^.

It is observed that the three aforementioned regions and the municipality of Trindade have similar sociodemographic profiles in terms of low income. This finding reaffirms the evidence of the study by Nery et al.^31^, which sought associations between social indicators and the detection of new cases of leprosy in Brazil, verifying that the incidence rates of the disease were directly related to socioeconomic and demographic conditions, with up to two times higher in populations with lower income and education levels^31^.

It is worth noting that the Sertão do São Francisco and Sertão do Araripe regions, with clusters of municipalities at high risk for the coefficients of occurrence and late diagnosis of leprosy, are border regions with the state of Ceará, Piauí and Bahia, states considered to be highly endemic^32–34^. Municipalities that have very high prevalences are usually surrounded by others that also have high or medium prevalences^35^. Thus, the municipalities that border Pernambuco can, through interstate migration, increase the transmission of leprosy, as with other infectious diseases in the country^36^.

The cluster of municipalities with the low/low pattern (Moran Map) for the three epidemiological indicators needs to be analyzed with caution, as these municipalities may be free of the disease or may reflect possible failures in health services, with underreporting of cases or the late diagnosis of infection^2,33^.

Such municipalities need to improve the diagnosis and treatment of the disease, as well as the surveillance and health education system, since leprosy control actions, when not performed with quality, can aggravate the risk of illness in certain regions^37^.

The use of spatial analysis in the area of public health has brought great advances in understanding the distribution of the disease, identifying areas of risk and silent areas, as well as possible operational problems, thus reducing costs and providing opportunities for a better assessment of the impact of public policies, directing actions consistent with the epidemiological reality^2^.

This study has limitations due to the use of secondary data from information systems, which may present inconsistency in the quantity, quality, and processing of their information, being subject to changes. This is relevant data, since each geographic area depends on its own technical-operational conditions of the epidemiological surveillance system to identify, notify, investigate, and confirm cases of leprosy.

To minimize such limitations, duplications, inconsistencies and incompleteness in the database were analyzed, with methodological rigor in the statistical procedures used, applying the smoothing of the Bayesian model to reduce random fluctuations.

In short, these findings contribute to reflections on leprosy control actions among managers, health professionals and the scientific community, since it was possible to identify priority areas for fighting the disease in Pernambuco.

It is emphasized, therefore, that further research is carried out to better understand the conditioning and determining factors related to this disease, as well as the probable disparities in the spatial detection coefficients of leprosy over the years in the state of Pernambuco.
